# Brain organoid: a 3D technology for investigating cellular composition and interactions in human neurological development and disease models in vitro

**DOI:** 10.1186/s13287-021-02369-8

**Published:** 2021-07-31

**Authors:** Oluwafemi Solomon Agboola, Xinglin Hu, Zhiyan Shan, Yanshuang Wu, Lei Lei

**Affiliations:** 1 Department of Histology and Embryology, Basic Medical Science College, Harbin Medical University, 194 Xuefu Rd, Nangang District, Heilongjiang Province Harbin, 150081 People’s Republic of China; 2 Key Laboratory of Preservative of Human Genetic Resources and Disease Control in China, Harbin Medical University, Ministry of Education, Harbin, China

**Keywords:** Brain organoid, Disease model, Cellular composition, Cellular interactions, Neurological development

## Abstract

**Abstract:**

The study of human brain physiology, including cellular interactions in normal and disease conditions, has been a challenge due to its complexity and unavailability. Induced pluripotent stem cell (iPSC) study is indispensable in the study of the pathophysiology of neurological disorders. Nevertheless, monolayer systems lack the cytoarchitecture necessary for cellular interactions and neurological disease modeling. Brain organoids generated from human pluripotent stem cells supply an ideal environment to model both cellular interactions and pathophysiology of the human brain. This review article discusses the composition and interactions among neural lineage and non-central nervous system cell types in brain organoids, current studies, and future perspectives in brain organoid research. Ultimately, the promise of brain organoids is to unveil previously inaccessible features of neurobiology that emerge from complex cellular interactions and to improve our mechanistic understanding of neural development and diseases.

**Graphical abstract:**

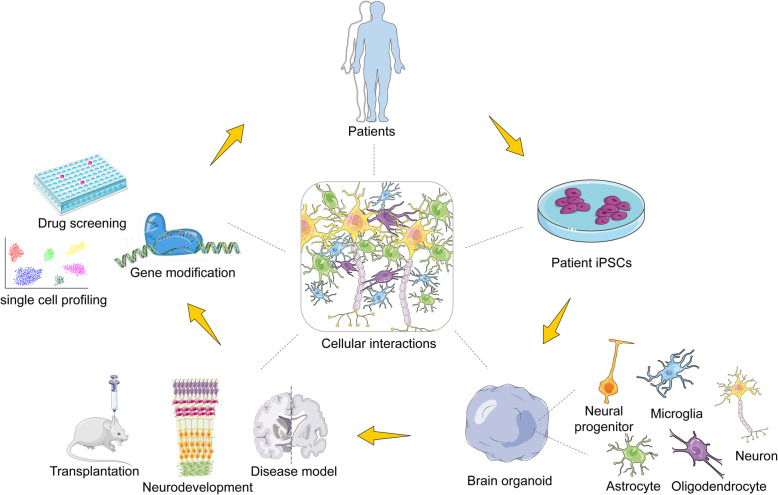

**Supplementary Information:**

The online version contains supplementary material available at 10.1186/s13287-021-02369-8.

## Introduction

**Fig. 1:**
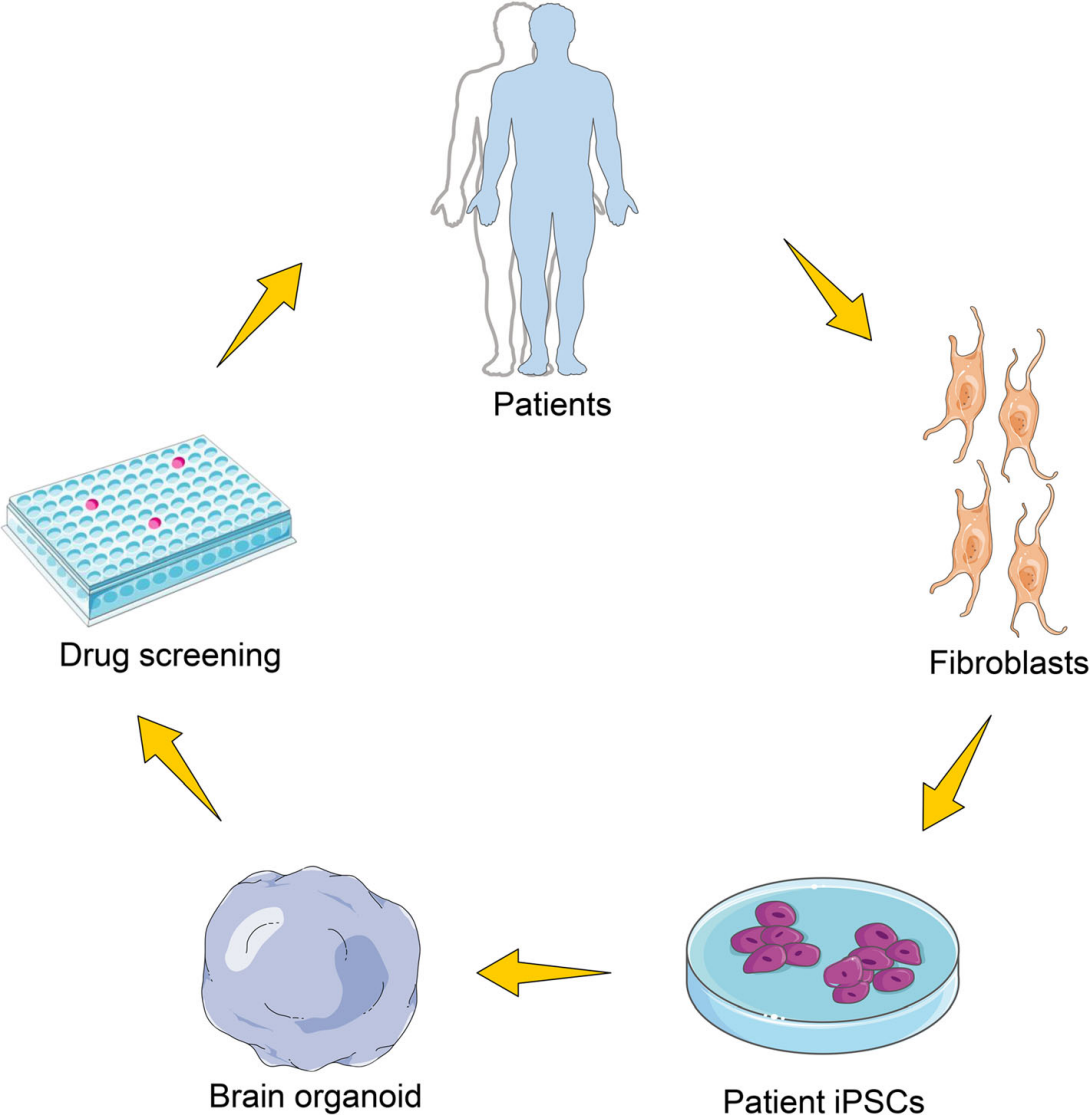
Brain organoid generation and therapeutic potential. Brain organoids can be generated from patient induced pluripotent stem cells (iPSCs) derived from adult fibroblasts and can be used to model human neurological disease. Drug screening could be one of potential applications by predicting drug efficacy before treatment of patients

Organoids are in vitro-derived structures that undergo some level of self-organization and resemble, at least in part, in vivo organs [1]. Organoid generation depends on the outstanding ability of stem cells to self-organize to sophisticated tissue structures. These self-organized structures may contain areas recapitulating different regions of the brain, often referred to as “brain organoids” or “cerebral organoids,” mirroring the broad presence of human brain regions in vivo. Alternatively, brain organoids may exhibit a structural phenotype that mimics specific brain regions, referred to as organoids of a specific region [2, 3]. The further development of our understanding of the development of the human nervous system and elucidation of the mechanisms that lead to brain disorders represent some of the most challenging ongoing endeavors in neurobiology. Therefore, the generation of region-specific three-dimensional (3D) models to study human brain development offers great promise for the study of the nervous system in both healthy individuals and patients [4]. Modeling of neuropsychiatric and neurological diseases with induced pluripotent stem cell (iPSC)-derived organoids could find application in the development of the new drugs (Fig. 1). Xenotransplanting of 3D organoids into mice unveiled the potential to restore the metabolic pressure caused by in vitro cell culture [5].

In light of the current available knowledge, in this review we discuss (i) cellular composition and interactions in brain organoids and (ii) the use of brain organoids as a disease model. The term “brain organoid” in used in reference to the entire structure; when referring to their specific studies, the terms used by authors are used.

## The development of 3D organoid system contributes to the reiteration of neural structure and cytoarchitecture

**Fig. 2:**
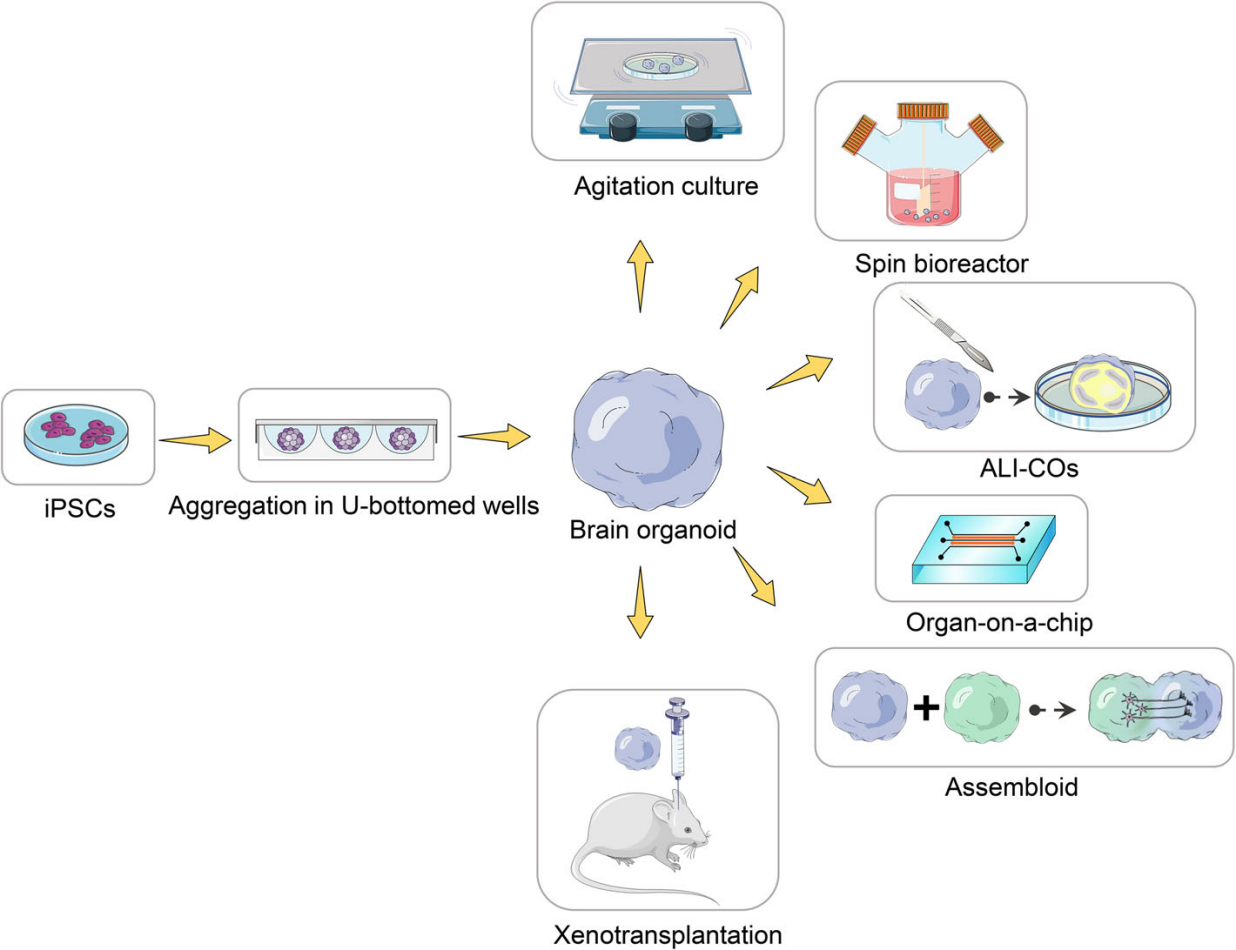
Major technical applications for culturing and analyzing brain organoids. Brain organogenesis could begin from embryoid bodies (EBs) generated from aggregates of iPSCs by centrifugation in U-bottomed wells. Brain organoids can be derived from EBs through undirected differentiation methods that lack inductive signals, or by patterning through directed methods to resemble specific brain regions (e.g., forebrain, midbrain, retina). These 3D cultures can be subsequently maintained by agitation culture, or spin bioreactor, or maintained in a multichannel microfluid chip. Brain organoids that resemble specific regions of the nervous system can be fused to generate brain assembloids. ALI-COs were maintained by organotypic slice culture at the air–liquid interface to improve oxygen supply, leading to improved neuronal survival and long-range projection formation. Transplanting brain organoids provides a strategy to establish a vascularized and functional in vivo model. The structure of functional neuronal networks and blood vessels in the grafts offers an unprecedented opportunity to model human brain development and disease

Technologies to derive brain organoids and assembloids that comprise certain neural structures are increasingly utilized to study cellular interactions between cell types in neural development and disease. Different organoid systems can be chosen for specific applications that could be used to generate specific neural structure, the desired cellular composition, and complex cellular interactions (Table 1). Pluripotent stem cells (PSCs) were differentiated, relying on intrinsic signaling and self-assembly towards the ectodermal germ lineage to generate brain organoids. This technique unveiled the different phases, namely embryoid body (EB) (Fig. 2), induction, expansion, and maturation phases [16]. Therefore, reproducibility and consistency are essential for the success of brain organoid protocols in a qualitative and quantitative system to model disease and for compound testing. Implantation of brain organoids into the mouse cortex led to an increased population of astrocytes, oligodendrocytes, and microglial cells and prolonged tissue survival contrary to the reports of organoids shrinking in size, probably due to progressive neuronal cell loss [6].

**Table 1:** Approaches in the establishment of 3D brain organoids

Approaches	Organoid type	Tissue structure	PROS	CONS	References
Xenotransplantation	Forebrain	Axonal projections, synaptogenesis mapping	Long-term culturing	Lack of vascular bed	Mansour et al., 2018 [6], Cakir, B. et al., 2019 [7], Wang, Z et al., 2020 [8]
Air–liquid interface-cerebral organoids (ALI-COs)	Whole brain organoid	Axonal tracts	Proper neural tract formation, long-term culturing	Devoid of vascularity	Giandomenico et al., 2019 [9]
Miniaturized spinning bioreactors	Forebrain, midbrain and hypothalamus	Defined oSVZ, and human oRGC-like NPCs, hypothalamic neurons	Patterning into different brain-like subregions, smaller volume of medium required, high reproducibility	Expensive for mass production and lacks vascularization	Qian, X et al., 2018 [10], Romero-Morales et al., 2019 [11]
Assembloids	Dorsal and ventral forebrain	Dorsal-ventral axis	Robust directional GABAergic interneuron migration, rough organization into cortical layers	Lack of output and input systems	Bagley et al., 2017 [3], Xiang, Y et al., 2019 [12]
Bioengineered scaffolds	Forebrain	Polarized cortical plate and radial units	Enhance tissue identity and architecture, and establish organoid models for teratogenic compounds Generation of patients’ specific disease-relevant cell types	Poor spatial orientation	Lancaster et al., 2016 [13], Sood et al., 2019 [14], Zafeiriou, et al., 2020 [15]

Although less extrinsic interference enables PSCs to follow their intrinsic program to grow and proliferate similar to in vivo, unavoidably, tissue heterogeneity arises, along with batch and iPSC line variability [10], making it cumbersome to control experimental conditions. To achieve a balance between intrinsic programming and stochasticity, hiPSCs were driven towards a homogeneous population of progenitors of specific brain regions (dorsal forebrain [17], ventral forebrain [3], midbrain [18] and hypothalamus [19]), and switched to promote tissue proliferation and expansion with fewer instructive signals. The culture of EBs in the spin bioreactor spinning system encourages proliferation and differentiation into brain organoids representative of specific brain regions. This technique is highly reproducible, improves efficiency, and reduced cost; however, it presents challenges such as lack of vascularization, risk of contamination, inability to control the number of neural-tube-like structures generated from each EB, and poorly generated organoids exhibiting irregular protrusions and crevices [10].

Interestingly, air–liquid interface (ALI) technique of culturing cerebral organoids led to improved neuronal survival (over 12 months) and axon outgrowth with various morphologies and active neuronal networks, which could innervate mouse spinal cord explants and evoke contractions of adjacent muscle tissue in a manner dependent on intact organoid-derived innervating tracts [9]. Air–liquid interface-cerebral organoids (ALI-COs) facilitate the study of neurodevelopmental conditions of the corpus callosum, neuronal circuit imbalances, and other defects where connectivity is thought to play a role. Examples include spinal cord injury, white matter stroke, amyotrophic lateral sclerosis [20], and the effects of environmental damage [21].

Recently, Xiang et al. designed a protocol to differentiate human embyonic stem cells (hESCs) to thalamic organoids that can specifically recapitulate thalamus development. Using a 3D system, the fusion of two distinct region-specific organoids predicted the interaction between developing thalamus and cortex [12]. Besides, brain organoid was generated using the one-stop microfluidic technique presenting advantages of a simplicity, scalability, and high reproducibility of the technique. This technique is suggested to be useful in culturing of other forms of organoid (liver, kidney, tumor, and eye), and therefore presents a useful tool in the study of organ development, developmental disorders, pharmacology and toxicology, and drug screening studies [22].

Despite their advantages, brain organoids lack stroma, tissue-resident immune cells, and in particular, vasculature, an essential niche during human brain development and disease conditions. Therefore, the generation of brain organoids that closely resemble in vivo tissue architecture requires the incorporation of connective tissue and tissue tissue-resident immune cells, as well as functional vasculature, which is essential in studying diseases [23]. Xenotransplantation of human brain organoids into the mouse brain revealed vascularization by the host and microglia migration into the brain organoid graft [10]. Although neural and stromal elements are not of the same species, generation and co-culturing of brain organoids and differentiated endothelial cells (ECs) from iPSCs of a UC Davis patient showed blood vessel generation, with capillary morphology inside the brain organoid and no signs of discontinuity on confocal imaging. However, blood vessels were not solely peripheral but penetrated their centre in xenotransplantation condition [24].

Blood vessel components (ECs, pericytes, and adventitial connective tissue) do not only receive contributions from ECs, but also from mesodermal progenitor cells, the angioblast, and the surrounding mesenchyme. The generation of human vascularized organoids byco-culturing mesodermal progenitor cells with mesodermal aggregates derived thereof resulted in tumor organoids with high uniformity in size, exhibiting a distribution of the endothelial network with high plasticity [23]. The generation of a neurovascular microenvironment similar to the in vivo situation by co-culturing neuroepithelium and embryonic mesenchyme revealed a defined interaction triggering the formation of the perineural vascular plexus [23]. Mesenchyme generates vasculature and hematopoietic cells. Therefore, early blood islands contribute to the microglia pool, although microglia naturally emerge from yolk sac blood islands [25]. Future studies are necessary investigate the generation of a uniformly vascularized structure with long-term connection to a circulatory system.

## Cellular composition of brain organoid

**Fig. 3:**
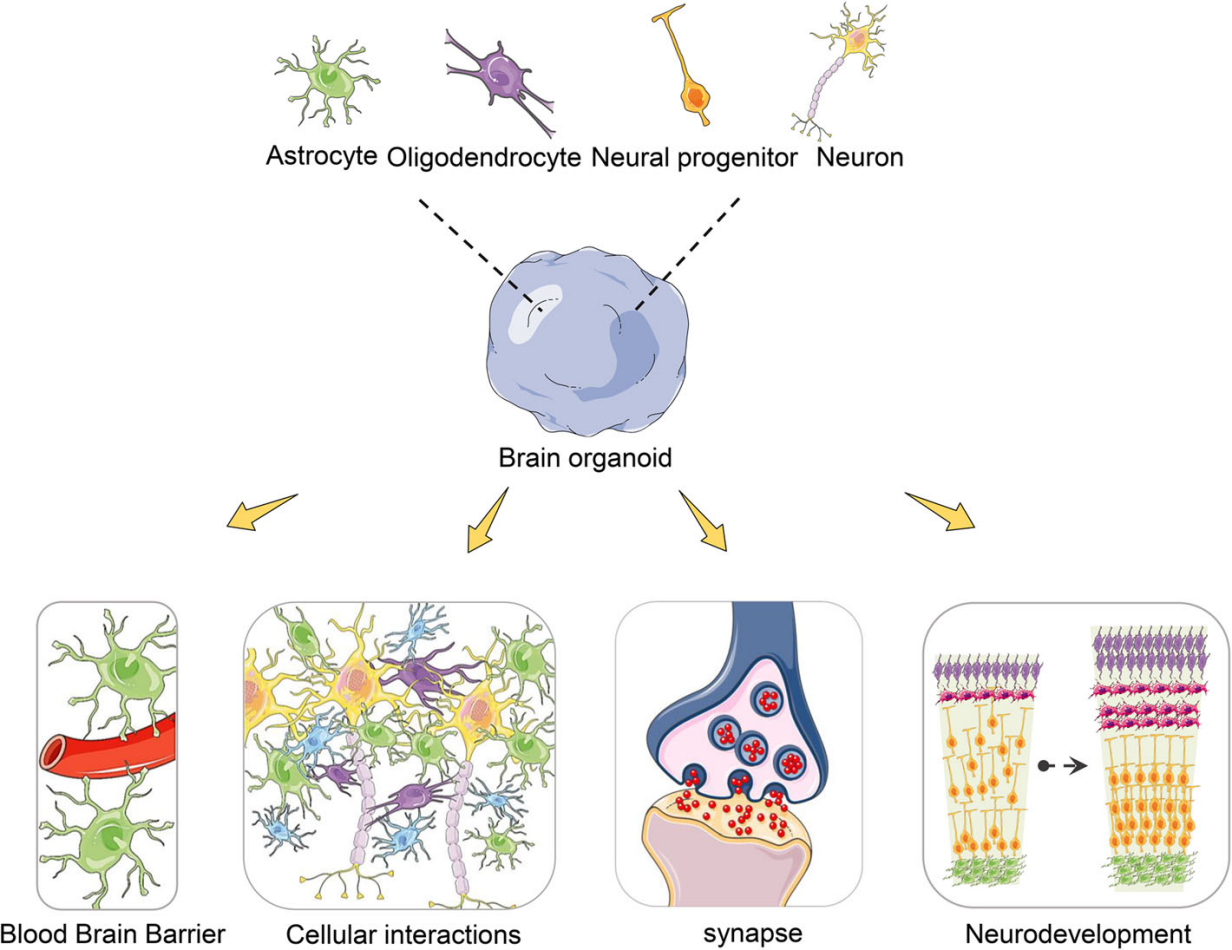
Modelling cellular composition and their interactions in brain organoid. Brain organoids comprise a great diversity of cell types, such as neural progenitors, neurons, astrocytes, and oligodendrocytes, which are organized into the same anatomical structures in developmental processes of the human brain. Brain organoids are increasingly utilized to model human neurodevelopment and occurrence and development of disease by studying the crosstalk between cell types in nervous system and non-central nervous system

The human brain is comprised of a great diversity of cell types from the neuroectodermal lineage (progenitors, neurons [about 250 neuronal sub-types [26], glial cells, oligodendrocytes, microglia, and vascular cells), mainly generated during embryonic development. Although brain organoids are plagued by high organoid-to-organoid variability, they comprise the same compendium of cell types (Table 2) which are organized into the anatomical structures in developmental processes of the human brain (Fig. 3) [42]. Upon the generation of radial glial cells (RGCs), intermediate progenitors (IPs), and deep- and superficial-layer neurons in an ordered temporal fashion, these different neuronal cell subclasses displayed multilaminar grouping, although they did not form the six distinct layers of the mammalian cortex and the anatomical location of cells can be a poor indicator of cell identity due to disorganization of organoid tissue [21, 43]. In brain organoids, generation of an outer subventricular zone (oSVZ) containing an abundant population of outer radial glia cell (oRGC) progenitor cells has been reported [2, 16].

**Table 2:** Summary for cellular composition and tissue type in brain organoid

Cell or tissue type	Organoid type	Days of differentiation	Characteristics	Sources	References
Neural progenitor cells	Dorsal forebrain	7–28 days	Located at ventricular zone	hiPSC	Lancaster & Knoblich, 2014 [27]; Qian et al.,2019 [28]; Wang, L et al., 2020 [29]
Glutamatergic neurons	Human cortical spheroid	15 weeks	Generated repetitive action potentials at depolarization. vGLUT	hIPSC	Yoon et al., 2019 [30]; Xiang et al., 2020 [31]; Zafeiriou, M et al., 2020 [15]
GABAergic neurons	Ventral forebrain	2 months	GABA, GAD67	hiPSC	Birey et al., 2017 [32]; Zafeiriou, M et al., 2020 [15]
Cortical interneurons	Forebrain assembloids	1–1.6 months post assembly	Migrate in a saltatory pattern and integrate into cortical microcircuit. SP8, GSX2	hiPSC	Birey et al., 2017 [32]; Xiang et al., 2017 [33]; Tanaka Y et al., 2020 [34]
Dopaminergic neurons	Midbrain	≥ 3 months	FOXA2, TH	hiPSC	Jo, J. et al 2016 [18]; Qian, X. et al., 2018 [10]; Smits L M et al., 2019 [17];
Hypothalamic/peptidergic neurons	Hypothalamus	≥ 3 months	Rax1, POMC, OTP	hiPSC	Qian, X. et al., 2018 [10]; Miura Y et al.,2019 [19]
Astrocyte	Asteroids	9–20 months	GFAP	hPSC	Sloan et al., 2017 [35]; Zafeiriou, M et al., 2020 [15]; Yakoub 2019 [36]
Microglia	Dorsal forebrain/organoid-grown microglia	13–52 days	IBA-1, IL34, CSF1, and TGFB1	Mesodermal progenitors	Ormel et al., 2018 [25]; Song, L et al., 2019a [37]
Oligodendrocyte	Oligocortical spheroids	5–8 months	OLIG2; MBP/CNP	hPSC	Madhavan et al., 2018 [38]; Zafeiriou, M et al., 2020 [15]
Optic vesicle (OV)-like structures	Retinal organoids	4–23 weeks	Possesses dense translucent projections at the apical edge that grow. OTX2, CRX	hiPSC-derived LCA4 patient	Lukovic et al., 2020 [39]; Brighi et al.,2020 [40]; Takata N et al., 2017 [41]

The characteristic mitotic behavior of oRGCs as observed by time-lapse confocal microscopy of organoid cells provided further evidence of their identity, and single-cell transcriptome sequencing was conducted to confirm the presence of cells with oRG gene expression signatures [44, 45]. A report described expansion and surface folding of cerebral organoids following phosphatase and tensin homolog (*PTEN*) deletion and enhancement of the phosphoinositide 3-kinase (PI3K)–AKT growth signaling pathway [46], suggesting that increased neural progenitor proliferation may be a major contributing factor to expansion and gyrification of the human brain. However, folding in the organoids was prominent in the neuroepithelium, and the relationship of this folding phenotype to actual cortical folding remains unclear. A 30-day organoid exhibited the presence of intermediate neurons and immature neurons; these neurons contribute to the formation of the precortical plate, while Cajal-Retzius cells play a vital role in the generation of the cortical plate architectural structure [43]. Mature neurons in brain organoids exhibited functionality neural activity, as evidenced by calcium dye imaging to detect calcium oscillations and synaptic formation [9]. The patterning of neural spheroids to the forebrain established human cortical spheroids and human subpallial spheroids; forming assembloids recapitulate the interactions of glutamatergic and GABAergic neurons seen in vivo. Astrocytes are also present in the human forebrain-specific spheroids and undergo maturation when the spheroids are cultured over 6 months [4, 6].

Microglia are resident histiocyte-type cells, constituting about one fifth of glial cells, which are homogeneously distributed; microglia are vital innate immune effectors of the central nervous system (CNS). Embryologically, microglia are derivatives of yolk sac cells that migrate to the developing brain, where they mature [47]. Microglia are separately generated from human iPSCs and then embodied into brain organoids to form multilineage assembloids, providing a suitable environment to study microglial morphology, migration, and response to injury [37, 48]. Another approach uses innately developed microglia within a cerebral organoid model that display their characteristic ramified morphology. The response of organoid-grown microglia (oMGs) to inflammatory stimulation mimics the response of adult microglia acutely isolated from post mortem human brain tissue [25].

## Interactions between neural lineage cells in organoids

Understanding of human brain development is essential for the study of cellular interactions within brain organoids. The neural tube in humans comprises apico-basally polarized neuroepithelial (NE) cells that surround a fluid-filled lumen. After initial lateral expansion, NE cells differentiate to RGC. The RGC later generates more differentiated cell types that migrate basally outward. Intermediate populations include IPs and basal RGC (bRG), or oRG [49, 50]. The bRG exhibits an expression profile that is relatively similar to that of the RGC [51], but shows heterogeneous morphologies [52], often lacking the apical connection. While the RGC maintains the cell body within a dense apical region called the ventricular zone (VZ), IPs and bRG translocate their cell bodies to a more basal territory termed the subventricular zone (SVZ) [53]. Once neurons are produced, they must migrate to their proper locations. Neurons generated within the cortex rely on the long basal fascicle of the RGC as a guide to translocate radially [54] from the VZ or SVZ through a cell-poor region termed the intermediate zone to find their final destination within the outer cortical plate.

## Progenitor cell interactions

Cortical cells are generated from neural progenitor cells (NPCs) situated in the VZ and SVZ [55]. NPCs, at the early stages of cortical formation, perform proliferation and differentiate towards neurons. Neurons migrate in an inside-out pattern to the developing cortex, acquiring their final phenotype. NEs are the first neural stem cell population to appear in the VZ of the newly developed neocortex [56]. In the early stages of neurogenesis, apical RGCs (aRGCs) proliferate to form the progenitor pool. Thereafter, they switch to asymmetric divisions generating either other NPC types, such as the SVZ basal progenitor cells, or neurons, which migrate away from the VZ and occupy the newly formed cortical plate [57]. In addition, aRGCs serve as a scaffold guiding the migration of newly formed neurons towards the developing cortical layers. However, aRGC fibers spread the whole depth of the developing cortex at the early stages of development; after gestational week 17 (GW17), they display truncated morphologies with their basal process terminating in the oSVZ [58].

Basal progenitor cells are a new class of progenitor cells; these cells lose apical contact with the ventricular surface and separates from the ventricles. In humans, during the GW11 and GW13, bRGs are one of the most abundant NPC populations in the developing cortex when the distinction of the oSVZ from the inner subventricular zone (iSVZ) occurs [49]. bRGs are a leading source of basal fibers; these fibers guide the migrating neurons, and secondly, bRGs self-amplify their number by performing several rounds of proliferative divisions [59]. The difference in gene profile and morphology among bRG cell types result in the great variety of neuronal types found in primates, thereby increasing the complexity of the developing cortex, and can also explain to some extent how some regions of the primate cortex evolve into gyri and others into sulci [60]. Capturing these interactions in human brain organoids allows the study of disorders linked to alterations in early neural development, such as Miller–Dieker syndrome.

## Neuron-neuron interactions

Neuronal interactions are the most frequently reported cellular interactions in brain organoids. Forebrain assembled demonstrated that glutamatergic neurons are able to generate synapses with GABAergic interneurons, similar to that seen in vivo and also that the reproducibility of brain organoids strongly depends on the hiPSC lines used and the experimental protocol [15]. Formed synaptic junctions of glutamatergic neurons and GABAergic interneurons are synaptically embodied into a microcircuit and increase morphological complexity [30, 32]. The saltatory migration of interneurons is better observed in brain organoids that in the 2D system. The assembled technique was recently employed to study the interaction of neurons between the cortex and thalamus [12]. Furthermore, neurons derived from organoids interacted not only with other organoid-derived neurons but as well with host neurons when xenotransplanted into the adult mouse cortex, revealing synaptic formation between host neurons and organoid neurons. Organoid-derived neurons in hosts have indeed been shown to differentiate, mature, and extend axonal projections posttransplantation [6, 61].

## Astrocytic-neuronal interactions

Astrocytes contribute vitally to the blood–brain barrier (BBB), exhibiting extensive neuronal interaction, thereby influencing neuronal development, maturation, synapse formation, and survival [62, 63]. RNA sequencing on hCS-purified astrocyte lineage cells at different time-points revealed astrocytic morphological changes, consistent with transcriptome-level evidence of maturation, and increases in astrocytic population and functionality over time were reported [35]. The interactions of human astrocytes (hAstros) and the high density of neurons in 3D tissue indicated complex astrocyte morphology. This interaction showed hAstros were distributed into territories, displaying club-like bulbous endings, and produced long-projecting varicose processes through the territories, similar to phenotypes observed in vivo. The report showed that in contrast to axons only, neuron cell bodies and dendrites promoted a more gray-matter-like appearance of hAstros. Astrocytes derived from human brain organoids were reported to generate synapses with neurons and to take up glutamate and synaptosomes, thereby increasing the amplitude of calcium dynamics in human neurons [64].

Brain organoid-derived astrocytes enhanced neurite growth and survival of mouse embryonic cortical neurons in a co-culture model with murine embryonic cortical neurons. Human astrocytes were isolated from BR1 and H9 cerebral organoids, these astrocytes were shown to have a phenotype similar to that of human adult astrocytes, exhibiting activity of the same 13 major representative pathways observed in adult human astrocytes, using proteomic assay. These astrocytes responded to an ATP stimulus with asynchronous calcium waves a hallmark of the biological functionality of astrocytes [65]. Unfortunately, live imaging of astrocytic-neuronal interactions remains a challenge; improvements in biotechnological methods and imaging instrumentation may help in establishing astrocytic-neuronal interactions in detail. Finally, it remains to be determined whether postnatal astrocytes are able to produce mature calcium responses in human neurons and whether this is due to direct cell-cell interactions or secreted factors.

## Microglial-neuronal interactions

In order to study phagocytosis, the release of chemokines and cytokines, synaptic junction formation, and removal of microglia in vitro, the generation of microglial in brain organoids is paramount [66]. In utero electroporation of 14–15-day-old embryos (E14–E15) to investigate microglia–dendrite interactions showed microglia–neuron contacts in 8–10-day-old pups, established in the developing somatosensory cortex. Microglia–dendrite contacts were frequently followed by the formation of new dendritic filopodia. Also, it revealed local neuronal calcium responses in dendrites following microglial contacts that were frequently followed by filopodia formation [67]. Mesodermal progenitors gave rise to microglia-like cells that physically interacted with neurons and were able to phagocytose synaptic structures in brain organoids [25].

Interestingly, oMGs at day 31 were located in close proximity to neurites upon further investigation, these oMGs and their processes became more intertwined with neuronal processes at day 52, and the distribution of microglia showed significant overlapping between IBA-1 and PSD-95 signals. In addition, the migratory behavior, calcium dynamics, and the response to proinflammatory stimuli of microglia differ in diverse regions of brain organoids [68]. However, improving microglia generation in brain organoid will allow analysis of the pathophysiology of disorders such as HIV-associated dementia, Alzheimer’s disease, and Parkinson’s disease.

## Oligodendrocyte-neuronal interactions

The study of oligodendrocytes using monolayer cell cultures presents the challenge of reduced myelination ability [69]. Earlier reports of brain organoid protocols were devoid of oligodendrocyte constituents. Recently, alternative protocols have been developed to generate oligodendrocytes in organoid cultures, providing interaction with neuronal processes and the formation of compact myelin [70].

Oligodendrocyte progenitor cells (OPCs) and myelinating oligodendrocytes in hPSC-derived cortical spheroids induce oligodendrocyte differentiation and ultimately myelination upon a timed exposure to defined oligodendrocyte lineage growth factors and hormones. Oligocortical spheroids (OCS) revealed concentric, but often unorganized, wrapping of human axons with multiple layers of uncompacted myelin, with further maturation and deep and superficial layer-marked neuron populations organized into distinct cortical layers with MYRF^+^ oligodendrocytes present at both layers. These axons formed a distinct layer adjacent to the deep-layer neurons, oligodendrocyte processes co-localized with neurofilament-expressing neuronal axons [71].

Human oligodendrocyte spheroids (hOLSs) contain oligodendrocytes, astrocytes, and neurons. Oligodendrocyte lineage cells mature transcriptionally. Investigation of electrophysiological maturation of oligodendrocytes using whole-cell current clamp recordings from bipolar and multipolar Sox10^+^ cells in slices of hOLSs exhibited interaction of oligodendrocyte processes and nearby axons and showed various stages of myelination, including lamellae of compact myelin surrounding axons [70].

Intriguingly, a report of an accelerated maturation of oligodendrocyte in brain organoids compared to the in vivo situation revealed a shorter culturing time. The resemblance of oligodendrocyte lineage cells in organoids mimics closely primary adult oligodendrocyte progenitor cells and myelinating oligodendrocytes following transcriptional profiling [32].

## Vascular cell interactions

Reports of brain organoid vascularization improved the quality of brain organoids (via nutrients transport, reduce apoptosis, and stress) and resemblance to the human brain [6, 23]. Endothelial cells and pericytes are major endodermal components of the brain vasculature, influencing the movement of molecules into the brain parenchyma from the blood.

The BBB is an essential component in the vascularization of the human brain. An attempt to model the BBB mimicking an in vivo environment that incorporated endothelial cells, astrocytes, neurons, and pericytes on a microfluidic platform to create a microphysiological system [72]. Another attempt was reported of xenotransplantation of brain organoids to the cortex of a mature mouse, resulting in the vascularization of brain organoid by the invasion of host blood vessels, allowing the interactions between the cells forming blood vessels and the surrounding brain organoid graft, demonstrating the development of functional vasculature networks inside the grafts [6]. In addition, at 90 days postimplantation, bundles of axons had grown out of the organoids through the cortical layers, and the corpus callosum, the amygdalar nucleus, and the striatum, as well as human axons with lower fiber density, were observed ipsilaterally in the hippocampus, hypothalamus, and thalamus, and in contralateral regions of the host brain. Grafted organoids did not only exhibit the ability to produce long-distance axonal projections to distant targets in the host brain but also possess the capacity to form synapses with host brain neurons. They showed synchronized activity, rather than sparse, isolated activity, suggesting an active neuronal network in the graft [6].

Endothelial-astrocytic interactions were investigated by analyzing vascularization through hPSC-derived blood vessel-like-structures along with human mesenchymal stem cells (hMSCs), incorporated into brain organoids. By fusion of cortical neural progenitor cell spheroids and endothelial cell spheroids, genes of cell-cell communications were highly expressed [68]. Interestingly, endothelial cells have also been generated in brain organoids by genetically modifying hPSCs to express ETV2, generating vasculature-like structures, expressing tight junction-related genes, and exhibiting trans-endothelial electrical resistance (TEER) [7]. The generation of the BBB in future protocols may consider the features of brain microvascular endothelial cells (BMECs), which could increase TEER expression due to tight junctions [73] in developing vascular structures.

## Brain organoids as a model for disease

**Fig. 4:**
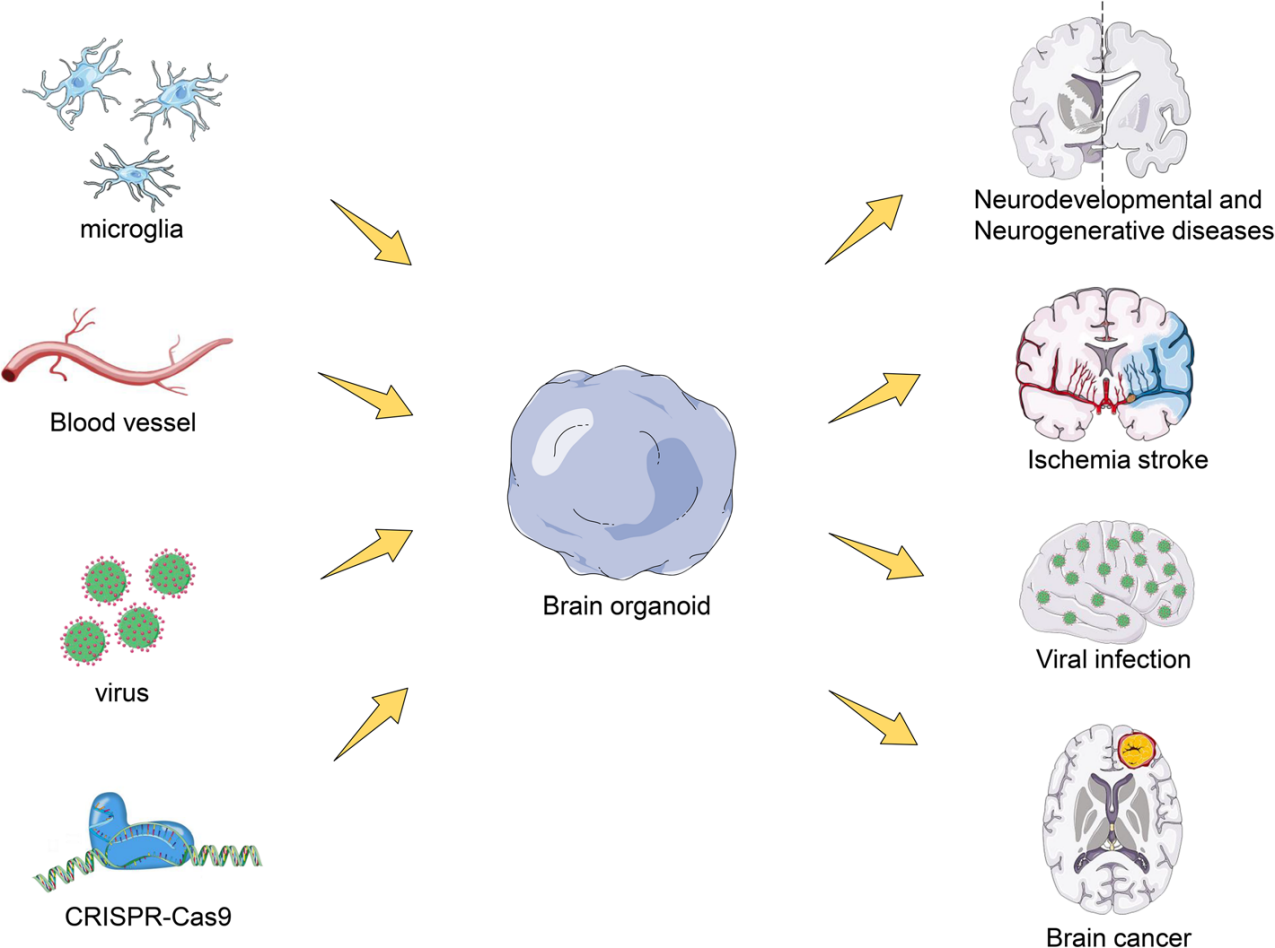
Brain organoids in disease modelling. Non-central nervous system-derived entities including microglia, blood vessels, and viruses can be added to brain organoids to model infected disease and vascular disease, which could help to study their interactions with cells in organoids. Brain organoids derived from patients or that are genetically engineered by CRISPR-Cas9 system to carry genetic variants associated with disease can be used to investigate disease pathogenesis in the nervous system

The environment best suited for disease studies would be an in vivo or samples directly isolated from patients. Unfortunately, these samples are often not readily available or their availability is restricted, subject to ethical approvals. However, brain organoids have provided insight and improved our sympathetic understanding of the developing human brain, but beyond this, studies involving brain organoids have also aimed at using the brain organoid to model disease conditions in humans (Fig. 4). In this session, we discuss (i) the roles of neural lineage cells and their interactions in brain-related disease that have been successfully modeled using brain organoids and (ii) examples of disease models with their respective phenotype (Table 3).

**Table 3:** Current application of cerebral organoids for cellular components and their interaction in disease model

Disease modelled	Organoid type	Cell type	Phenotype	References
Timothy syndrome	Assembloids (dorsal and ventral forebrain)	GABAergic interneuron	Abnormal saltation frequency and shorter saltation length	Birey et al., 2017 [32]
Miller–Dieker syndrome	Forebrain	Ventricular zone radial glial cells (vRGCs)	Decreased neuroepithelial loops with distorted cortical niche, abnormal vRG cell division, reduced size of organoids	Iefremova et al., 2017 [74], Bershteyn, M. et al., 2017 [44], Karzburn et al., 2018 [75]
Zika virus infection (ZIKV)	Forebrain	Neuronal progenitors	Smaller size organoids with larger ventricular lumen and reduced neuronal cell-layer thickness, increase ZIKV-induced cell apoptosis	Qian et al., 2017 [76], Xu, Y et al., 2019 [77]
Cytomegalovirus infection (CMV)	Dorsal forebrain	Neuronal progenitors	Decreased cellular proliferation, marred cortical lamination necrosis, induced-vacuolar and cystic degeneration	Brown, R. M. et al., 2019 [78], Sun, G. et al., 2020 [79]
Herpes simplex virus (HSV)	Neurosphere	Neurons	Vulnerability of matured neurons (MAP2 +) to destruction via lysis of HSV-1	D’Aiuto et al., 2019 [80]
Autism spectrum disorder	Dorsal forebrain	GABA/Glutamate neuron	Increased generation of NPCs and GABAergic neurons, overexpression of FOXG1	Mariani et al., 2015 [81], Wang, P. et al., 2017 [82]
Alzheimer’s disease (AD)	Dorsal Forebrain	Neurons	Induced amyloid aggregation, hyperphosphorylated tau protein and endosome abnormalities	Raja et al., 2016 [83], Pavoni, S. et al., 2018 [84], Papaspyropoulos, A. et al., 2020 [85]
Tuberous sclerosis complex (TSC)	Forebrain	Neuronal progenitors, neurons	A strong bias astro-glial fate generation, altered cellular morphology, activation of Mtorc1 signaling	Blair, J. D.et al., 2018 [86]

## Diseases related to virus infection

### Microcephaly and ZIKV

The modeling of microcephaly using brain organoids from fibroblast-derived iPSCs of microcephaly patients revealed the relatively smaller size of brain organoids compared to normal brain organoids, which serves as a control. The reduced size was suggestive of premature differentiation in neural progenitor regions [43]. Recently, scientific progress uncovered that asparaginyl-tRNA synthetase1 (NARS1) plays a significant role in microcephaly. Patient-derived organoid show reduced proliferation of RGCs, leading to smaller organoids [29]. In addition, microcephaly is suggested to be a result of viral infections [87]. Zika virus (ZIKV) infection caused a reduction in the size of brain organoids, with the virus targeting regions rich in NPCs initiating premature differentiation of NPCs, consistent with a previous report [21]. The activation of the innate immune receptor Toll-like receptor-3 (TLR3) by ZIKV infection caused cellular dysregulation and apoptosis; this phenotype was reversed when TLR3 was inhibited [88].

Most ZIKV cases are asymptomatic but associated with severe neurological abnormalities include Guillain-Barre syndrome, microcephaly [89]. The importance of 3D cultures in investigating the mechanisms inducing NPC death and reducing the overall growth of organoids upon ZIKV infection have been revealed. ZIKV exhibits a unique tropism towards SOX2^+^-NPCs and causes a decrease in neuronal cell-layer volume, resembling microcephaly- and lissencephaly-like phenotype in brain organoids [46, 76, 87]. ZIKV also disturbs centrosome function by promoting an incorrect orientation of the mitotic plane, resulting in NPC depletion, as has been reported in human neurospheres [90]. AXL receptor tyrosine kinase is considered a vital virus receptor in NPCs in 2D- and 3D-based studies. Interestingly, genetic ablation of AXL does not protect human NPCs and cerebral organoids from ZIKV [91], whereas inhibitors of AXL (R428) reduced the effects of virus-induced disruption in neurogenesis [92]. JMX0207 inhibited the interaction between NS3 and NS28, increasing antiviral efficiency and significantly reducing the effects of ZIKV infection on 3D brain organoids and animal models [93].

### Herpes simplex virus (HSV) and cytomegalovirus (CMV)

Brain organoids were reported to model both herpes simplex virus (HSV) and cytomegalovirus (CMV) infections in vitro. Infection of HSV-1 in brain organoids to study HSV-1 latency and reactivation in brain organoids revealed the vulnerability of matured neurons to destruction via lysis of HSV-1. Consistent with animal model studies, brain organoids showed difficulty in the reactivation of HSV-1 in the central nervous system, in contrast to monolayer study [80].

In addition, brain organoids from CMV-infected iPSCs revealed reduced valid cell count and increased number of cysts and vacuoles and necrosis. Aberrant β- tubulin III expression is associated with a disruption of neural projections and lamination within the cortical layers; these phenotypes are consistent with observations of human patient samples [94, 95]. However, brain organoid studies are not a complete substitute for in vivo studies of HSV and CMV due to the current limitations of differentiation of immune and inflammatory cells within the brain organoid architecture. Nevertheless, brain organoids have been able to recapitulate aspects of host-pathogen interaction in HSV and CMV infections.

## Neurodevelopmental and neurogenerative diseases

### Autism spectrum disorders (ASD)

Brain organoids have been modeled along with autism spectrum disorders (ASD), and the transcriptome of ASD has been compared with a control dataset of postmortem human brain transcriptomes from embryonic age to adulthood [96]. iPSCs alone with a CRISPR-cas9-induced heterozygote mutation of CDH8 have been used to model a form of non-idiopathic ASD in brain organoids, revealing upregulated expression of genes involved in differentiation of GABAergic neurons [82]. Similarly, brain organoid models with hyperactive mTOR were generated by deleting RAB39b, inducing the formation of enlarged organoids due to proliferation of NPCs and GABAergic neurons, consistent with an increased presence of a specific sodium channel isoform in ASD organoids [81, 97]. ASD organoids showed upregulation of FOXG1, an important regulator of forebrain differentiation linked to ASD-like neurodevelopmental syndromes [92]. However, the inhibition of FOXG1 by shRNA reverses the abnormally abundant GABAergic neurons [81]. iPSC-derived organoids from Angelman syndrome patients exhibited early silencing of paternal UBE3A, and topoisomerase inhibitors can rescue the protein level of UBE3A [98]. These reports are suggestive of how brain organoid coupled with gene editing techniques may be explored to study the molecular basis of genetically heterogeneous disorders and distorted dynamics in idiopathic ASDs.

### Diseases related to ASD

Rett syndrome (RTT) is a non-inherited disease caused predominantly by mutations in the X-linked gene methyl-CpG-binding protein 2 (MECP2) encoding epigenetic regulator [99]. The interaction between MECP2 and microRNA was studied in RTT patient-derived iPSCs, revealing upregulation of miR199 and miR214 and associated modulation of extracellular signal-regulated kinase (ERK) and AKT signaling pathway [100]. Brain organoid modeled in RTT exhibited an increase in the expansion of VZ as a result of increased NPC proliferation, although neurogenesis and neural maturation were disabled [101].

Timothy syndrome (TS) is a rare autosomal-dominant disorder caused by mutation of the l-type calcium channel (CACNA1C). Normal cortical development requires coordinated interaction between GABA interneurons and glutamatergic neurons [102, 103]. TS was modeled in brain organoid from iPSC-derived assembled recreated an environment for the integration of GABA interneurons into functional microcircuits to allow a leaping migration into the cortical plate as observed in vivo [32]. However, TS exhibited a distorted saltatory pattern, culminating in delayed neuronal migration, and also showed increased residual calcium in response to depolarization and the presence of functional synapsis between neural networks [104]. The blockage of CACNA1C using nimodipine or roscovitine re-established phenotypes of diseased assemblies [32].

### The Miller–Dieker syndrome (MDS)

MDS is a contiguous gene deletion syndrome of chromosome 17p13.3, characterized by abnormally smooth cortical surface and lack of folding structure in the brain (lissencephaly), as well as microcephaly. The smoother the cortical surface, the more severe associated symptoms are [105]. Brain organoids generated from iPSC-derived from MDS patients unveiled asymmetric phenotype of the RGCs of the VZ and the distortion of cytoskeletal (microtubule) structure of RGCs. Also, increased apoptosis of progenitor cells, limited migration of neurons, and an extended time of the mitotic process of oRGCs were observed [44, 74].

### Aicardi–Goutieres syndrome (AGS)

AGS is a rare autosomal recessive encephalopathy as a likely result of mutations in three-prime repair exonuclease 1 (TREX1), characterized by acquired microcephaly, cerebral calcification, leukodystrophy, and cerebral atrophy [103]. Brain organoids built using iPSCs deficient in TREX1 and/or organoids co-cultured in astrocyte devoid of TREX1-preconditioned medium led to reduced size and increased neuronal apoptosis [106]. The effects of astrocyte dysfunction on neurons in AGS remain unclear.

### Vanishing white matter disease (VWML)

Brain organoids were developed to mimic myelin formation, or interactions between oligodendrocytes and other cell types of the brain. Studies suggest that oligodendrocytes develop abnormally due to altered astrocyte development [107]. An environment for the co-culturing of astrocyte and oligodendrocytes in a single microunit which may be provided by brain organoid may help in solving mysteries related to vanishing white matter leukodystrophy.

### Tuberous sclerosis complex (TSC)

Tuberous sclerosis complex (TSC) is a rare multi-systemic autosomal-dominant genetic disease caused by activation of mammalian target of rapamycin (mTOR) complex by mutation of *TSC1* and *TSC2* genes, characterized by benign tumor growth [108]. Brain organoids can be used to model TTSC by CRISPR-cas9 editing in hESCs and by using patient-derived iPSCs mutant for *TSC1*and *TSC2* [86]. The reaction of the cellular phenotype of TSC in brain organoids was achieved by the deletion of the *TSC1* gene and heterozygosity of *TSC2*. However, the combined deletion of *TSC1* and *TSC2* did not result in abnormal proliferation and differentiation of neurons or glial cells, whereas brain organoids with either of *TSC1* and *TSC2* displayed a phenotype similar to human patients [109].

## Neurodegenerative disease

### Alzheimer’s disease (AD)

The application of brain organoids in modeling AD showed great promise in exhibiting amyloid-β deposition and hyperphosphorylation of tau protein [85, 110, 111]. An AD phenotype occurred as the organoid advanced in age, and treatment with β- or γ-secretase inhibitor reduced the level of amyloid-β deposition and hyperphosphorylation of tau protein in brain organoid [112, 113]. Recently, evidence has pointed towards glial cell dysfunction in association with AD pathogenesis. Using organoid biotechnology, hiPSC-derived neural cells were used for therapeutic target identification and as a relevant platform for drug screening in AD [114].

### Parkinson’s disease (PD)

Parkinson’s disease (PD) a long-term neurodegenerative disease that affects mainly the motor system. The neurodegeneration of dopaminergic neurons (DA neurons) located at the midbrain is the critical pathophysiology of PD [18]. A PD model was generated using midbrain organoids from leucine-rich repeat kinase 2 (*LRRK2*)-mutant iPSCs, characterized by a declined in DA neuron and mature neuron marker expression and abnormal increase in co-localization of α-synuclein and thioredoxin-interacting protein (TXNIP) [115]. Using a PD-specific PARK7-linked in vitro model, it was identified that U1 splice-site mutations are enriched in sporadic PD patients [116].

## Future research directions

The complexity of the human brain is associated with diverse cellular constitute and sophisticated interactions between cells and brain regions. The development of brain organoids has contributed immensely to our understanding of the functional interactions in the brain down to histological levels. However, at present, several challenges are associated with the generation of a brain organoid that closely mimics the human brain; one of such challenges is the generation of anterior-posterior and dorsal-ventral axis [3, 32]. Researchers have attempted to mimic this axis by generating assemblies, but this strategy is not ideal for mimicking the spatiotemporal axes; an alternative idea could be the microfluidic system [117] to produce the spatiotemporal concentration gradients. Interestingly, interactions between cortical neurons and thalamus have been established in hESC-derived thalamic organoids when fused with cortical organoids [12]; it will be exciting to see if future studies will consider cellular interactions in basal ganglia and the midbrain.

Furthermore, the generation of brain organoids is faced with the difficulty of having to be able to locate certain cell types and differentiate between the cell-autonomous and non-cell-autonomous effects in organoids as a result of a variety of cellular constitute generated in brain organoids [118]. Indirect protocols employed the generation of forebrain organoids patterned with a Sonic Hedgehog (SHH) gradient to investigate positional identities of internal cellular structures in forebrain organoids and assemblies. Further research is needed to investigate whether many brain regions could be assembled as an in vitro representative of the human brain and if these assemblies can be maintained over a prolonged time.

Irrespective of the challenges encountered in the production of brain organoids, a protocol had been reported for the generation of brain organoids to mimic the human fetal brain at the mid-gestational stage [2]. Recently, a study using electroencephalography showed the similarity between brain organoids and preterm neonatal brains, which is indicative that the development of structured network activity in a human neocortex model may follow stable genetic programming [119]. However, brain organoids are a vital foundation for the study of cellular interaction and circuit dysfunction, allowing for the investigation of (i) the pathogenetic roles of disease-causative mutations and (ii) potential therapeutic compounds through genetically manipulated brain organoids. Moreso et al. [35] reported the culturing of brain organoids for 20 months. It is expedient that brain organoids be cultured for a much longer period to generate mature cells to mimic the cellular composition of the adult human brain.

In addition, brain organoids are important alternative platforms in drug screening with high-throughput approaches [120]. For example [121], Hou et al. reported the development of primary pancreatic organoid tumor models for high-throughput phenotypic drug screening in modeling epithelial cancers. Moreover, high-throughput automation enhances kidney organoid differentiation from hPSC and enables multidimensional phenotypic screening [122]. Additionally, a team of researchers proposed the use of iPSC-derived organoids as a feasible tool for probing neurological complications of SARS-CoV-2 [123]. In all of these reports, well-established and validated protocols in exploiting brain organoids as an efficient drug screening tool remain lacking.

Finally, in addition to promoting cellular maturation and sustainability, organoid analysis tools (single-cell profiling [124], cutting-edge imaging, and functional interrogation methods) need to be enhanced; this will improve the provision of assays to facilitate disease modeling studies. Collectively, patient-derived organoids, assembloids, and technological advances in cell biology will uncover the cellular interactions in human neural development and diseases.

## Supplementary Information

**Additional file 1.**

## Data Availability

Not applicable as “Brain organoid: A 3D technology for investigating cellular composition and their interactions in human neurological development and disease models in vitro” is a “Review Article”.

## Abbreviations

iPSCs: Induced pluripotent stem cells
3D: Three-dimensional
PSCs: Pluripotent stem cells
EB: Embryoid body
ALI: Air–liquid interface
hESCs: Human embyonic stem cells
ALI-COs: Air–liquid interface-cerebral organoids
ECs: Endothelial cells
RGCs: Radial glial cells
IPs: Intermediate progenitors
oSVZ: Outer subventricular zone
oRGCs: Outer radial glia cells
PTEN: Tensin homolog
PI3K: Phosphoinositide 3-kinase
CNS: Central nervous system
oMGs: Organoid-grown microglia
NE: Neuroepithelial
bRG: Basal RGC
VZ: Ventricular zone
SVZ: Subventricular zone
NPCs: Neural progenitor cells
GW17: Gestational week 17
iSVZ: Inner subventricular zone
MDS: Miller–Dieker syndrome
BBB: Blood–brain barrier
hAstros: Human astrocytes
E14-E15: 14–15-day-old embryos
OPCs: Oligodendrocyte progenitor cells
OCS: Oligocortical spheroids
hOLSs: Human oligodendrocyte spheroids
TEER: Trans-endothelial electrical resistance
BMECs: Brain microvascular endothelial cells
NARS1: Asparaginyl-tRNA synthetase1
ZIKV: Zika virus
TLR3: Toll-like receptor-3
HSV: Herpes simplex virus
CMV: Cytomegalovirus
ASD: Autism spectrum disorders
RTT: Rett syndrome
TS: Timothy syndrome
CACNA1C: l-type calcium channel
MDS: The Miller–Dieker syndrome
AGS: Aicardi–Goutieres syndrome
TREX1: Three-prime repair exonuclease 1
VWML: Vanishing white matter disease
TSC: Tuberous sclerosis complex
AD: Alzheimer’s disease
PD: Parkinson’s disease
LRRK2: Leucine-rich repeat kinase 2
TXNIP: Thioredoxin-interacting protein
SHH: Sonic Hedgehog
