# Supplementary material for: Brain organoid: a 3D technology for investigating cellular composition and interactions in human neurological development and disease models in vitro
Source: Stem Cell Res Ther. 2021 Jul 31;12:430. doi: 10.1186/s13287-021-02369-8 (PMC8325286; doi:10.1186/s13287-021-02369-8)
Supplement: Supplementary file 1 — Additional file 1. [file 13287_2021_2369_MOESM1_ESM.html]

xml version="1.0" encoding="utf-8"?CEFirst Log 

|  |
| --- |
| CEFirst Log |
| Version : 8.4.3 |
| USER | DESKTOP-V2FTEVJ |

---

# Hyphenated Words

Please check the following hyphenated words for consistency:

- Air-liquid [hyphen 2, N-dash 5]
- autosomal-dominant [hyphen 1, spaced 1]
- Blood-brain [hyphen 3, N-dash 1]
- Brain-region [spaced 3, hyphen 1]
- co-culture [hyphen 1, closed-up 1]
- long-term [hyphen 3, spaced 1]
- micro-physiological [hyphen 1, closed-up 1]
- Miller–Dieker [N-dash 2, hyphen 5]
- neural-tube [hyphen 1, spaced 1]
- Organoid-grown [hyphen 2, spaced 1]
- organoid-to [hyphen 1, spaced 1]
- region-specific [hyphen 4, N-dash 2]
- single-cell [hyphen 3, spaced 1]
- Spatio-temporal [closed-up 3, hyphen 1]
- superficial-layer [hyphen 1, spaced 1]
- a-chip [hyphen 1]
- Aicardi–Goutieres [N-dash 2]
- Aicardi-Goutières [hyphen 1]
- ALI-COs [hyphen 4]
- Alves-Leon [hyphen 1]
- amyloid-β [hyphen 2]
- anterior-posterior [hyphen 1]
- apico-basally [hyphen 1]
- ASD-like [hyphen 1]
- Asparaginyl-tRNA [hyphen 2]
- astrocyte-secreted [hyphen 1]
- Astrocytic-neuronal [hyphen 3]
- astro-glial [hyphen 1]
- barrier-on [hyphen 1]
- blood-central [hyphen 1]
- Brainer-Lima [hyphen 1]
- brain-like [hyphen 2]
- brain-related [hyphen 1]
- byco-culturing [hyphen 1]
- Caceres-Palomo [hyphen 1]
- Cajal-Retzius [hyphen 1]
- cell-autonomous [hyphen 3]
- cell-cell [hyphen 2]
- cell-derived [hyphen 3]
- cell-layer [hyphen 2]
- cell-poor [hyphen 1]
- cells-derived [hyphen 1]
- Chandler-Militello [hyphen 1]
- club-like [hyphen 1]
- CMV-infected [hyphen 1]
- co-cultured [hyphen 1]
- co-culturing [hyphen 3]
- co-expression [hyphen 1]
- co-localization [hyphen 1]
- co-localized [hyphen 1]
- cutting-edge [hyphen 1]
- cytomegalovirus-induced [hyphen 1]
- day-old [hyphen 3]
- deep-layer [hyphen 1]
- disease-causative [hyphen 1]
- disease-relevant [hyphen 1]
- dorsal-ventral [hyphen 3]
- Endothelial-astrocytic [hyphen 1]
- fibroblast-derived [hyphen 1]
- fluid-filled [hyphen 1]
- forebrain-specific [hyphen 1]
- Garcia-Leon [hyphen 1]
- gray-matter [hyphen 1]
- Guillain-Barre [hyphen 1]
- hCS-purified [hyphen 1]
- hESC-derived [hyphen 2]
- high-throughput [hyphen 5]
- hiPSC-derived [hyphen 2]
- histiocyte-type [hyphen 1]
- HIV-associated [hyphen 1]
- host-pathogen [hyphen 1]
- hPSC-derived [hyphen 2]
- human-specific [hyphen 1]
- induced-vacuolar [hyphen 1]
- inside-out [hyphen 2]
- interface-cerebral [hyphen 3]
- intra-tumoral [hyphen 1]
- iPSC-derived [hyphen 10]
- iPSCs-derived [hyphen 1]
- junction-related [hyphen 1]
- layer-marked [hyphen 1]
- Leucine-rich [hyphen 2]
- lissencephaly-like [hyphen 1]
- long-distance [hyphen 1]
- long-projecting [hyphen 1]
- long-range [hyphen 1]
- l-type [hyphen 2]
- Mejias-Ortega [hyphen 1]
- methyl-CpG [hyphen 1]
- microglia–dendrite [N-dash 2]
- microglia-like [hyphen 2]
- Microglial-neuronal [hyphen 1]
- microglia–neuron [N-dash 1]
- midbrain-like [hyphen 2]
- mid-gestational [hyphen 1]
- mini-bioreactors [hyphen 1]
- mis-splicing [hyphen 1]
- multi-systemic [hyphen 1]
- neurofilament-expressing [hyphen 1]
- neuromelanin-producing [hyphen 1]
- Neuron-neuron [hyphen 1]
- non-cell [hyphen 2]
- non-central [hyphen 3]
- non-idiopathic [hyphen 1]
- non-inherited [hyphen 1]
- non-surface [hyphen 1]
- Oligodendrocyte-neuronal [hyphen 1]
- one-stop [hyphen 2]
- organoid-based [hyphen 1]
- organoid-derived [hyphen 4]
- oRGC-like [hyphen 1]
- pathway-dependent [hyphen 1]
- patient-based [hyphen 1]
- Patient-derived [hyphen 5]
- PD-specific [hyphen 1]
- re-established [hyphen 1]
- Romero-Morales [hyphen 1]
- Saia-Cereda [hyphen 1]
- Sanchez-Mejias [hyphen 1]
- Sandoval-Espinosa [hyphen 3]
- SARS-CoV [hyphen 2]
- self-amplify [hyphen 1]
- self-assembly [hyphen 1]
- self-organization [hyphen 2]
- self-organize [hyphen 1]
- self-organized [hyphen 2]
- Self-organizing [hyphen 1]
- signal-regulated [hyphen 1]
- species-specific [hyphen 1]
- splice-site [hyphen 1]
- sub-types [hyphen 1]
- surface-dividing [hyphen 2]
- system-derived [hyphen 1]
- Thioredoxin-interacting [hyphen 2]
- Three-dimensional [hyphen 6]
- Three-prime [hyphen 2]
- time-lapse [hyphen 1]
- time-points [hyphen 1]
- tissue-resident [hyphen 2]
- Toll-like [hyphen 2]
- transcriptome-level [hyphen 1]
- Trans-endothelial [hyphen 2]
- Twenty-five [hyphen 1]
- U-bottomed [hyphen 1]
- vascular-like [hyphen 1]
- vasculature-like [hyphen 1]
- vessel-like [hyphen 1]
- virus-induced [hyphen 2]
- vitro-derived [hyphen 1]
- well-established [hyphen 1]
- whole-cell [hyphen 1]
- Wynshaw-Boris [hyphen 1]
- X-linked [hyphen 1]
- ZIKV-induced [hyphen 1]
- α-synuclein [hyphen 1]
- γ-secretase [hyphen 1]

---

Copyright © 2013-2021 SPi Global, Chennai, India
